# Dietary Intake and Type 2 Diabetes

**DOI:** 10.3390/nu11092177

**Published:** 2019-09-11

**Authors:** Omorogieva Ojo

**Affiliations:** School of Health Sciences, University of Greenwich, London SE9 2UG, UK; o.ojo@greenwich.ac.uk; Tel.: +44-020-8331-8626; Fax: +44-020-8331-8060

**Keywords:** type 2 diabetes, dietary intake, glycaemic control, dietary management approaches, micronutrients, macronutrients, nutrition, chronic conditions, lipid parameters

## Abstract

This editorial aims to examine the risk factors associated with type 2 diabetes and to discuss the evidence relating to dietary strategies for managing people with this condition. It is clear from the evidence presented that a range of dietary interventions can provide useful approaches for managing people with type 2 diabetes, including the regulation of blood glucose and lipid parameters, and for reducing the risks of acute and chronic diabetic complications.

Diabetes is a metabolic condition that is characterized by chronic hyperglycemia and results from an interplay of genetic and environmental factors [1,2,3,4]. Its prevalence is on the increase in the UK and worldwide, partly due to changes in lifestyle that predispose individuals to obesity and being overweight [1,3,4,5,6]. It is estimated that about 90% of adults currently diagnosed with diabetes have type 2 diabetes and, based on a World Health Organisation (WHO) report, about 422 million adults were living with diabetes in 2014 compared with 108 million in 1980 and this condition caused about 1.5 million deaths in 2012 [7,8]. The United States of America has about 30.3 million adults living with diabetes, and 1.5 million estimated new diabetes cases are diagnosed every year, representing an increasing prevalence of this condition [9]. Diabetes presents a major public health challenge despite the developments in technology and the pharmaceutical industry [9]. These problems may be in the form of acute or chronic complications and changes in body composition can be profound. In this regard, Almusaylim et al. [10] conducted a cross-sectional and longitudinal study to evaluate the associations between variations in glycaemic status and changes in total body, trunk, appendicular fat mass, and lean mass in men. The longitudinal analyses demonstrated that changes in total body, fat mass and lean mass, and appendicular lean mass differed among glycaemic groups [10]. In addition, glucose dysregulation was found to be related to adverse changes in total body and appendicular lean mass.

Therefore, in order to attenuate the problems of diabetes, management strategies usually include lifestyle changes such as increased physical activities and dietary interventions. Studies that evaluate the role of nutrition in the management of type 2 diabetes often involve human and animal models as these approaches enable us to have a broader and more in-depth understanding of the condition. Sometimes, diabetes may co-exist with other conditions such as stroke and these may present unique challenges in relation to nutritional interventions.

The current editorial aims to evaluate the risk factors associated with type 2 diabetes and the role of diet in the management of people with the condition. It involves evidence drawn from human and animal studies.

In one of the studies, Muñoz-Garach et al. [11] examined the role of vitamin D status, calcium intake, and the risk of developing type 2 diabetes. According to the authors, the role of vitamin D in glucose homeostasis appears to be its association with insulin secretion, insulin resistance, and systemic inflammation and this is one of its important non-skeletal functions [11]. In addition, there seems to be a link between the consumption of dairy products and a lower risk of type 2 diabetes and this has been demonstrated in many observational studies although the mechanism and the role of calcium intake in the risk of developing this condition have not been well established [11]. Therefore, a randomized controlled trial on the role of vitamin D and calcium in the development of type 2 diabetes will further elucidate our understanding of the mechanisms of action of these micronutrients [11]. In a related study, Contreras-Manzano et al. [12] explored cardiovascular risk factors and their association with vitamin D deficiency based on a nationally representative sample of 3260 young Mexican women. The authors found that the prevalence of vitamin D deficiency among the women aged 20 to 49 years old was a public health problem and that obesity, type 2 diabetes, and high total cholesterol were found to be associated with vitamin D deficiency.

On the other hand, Fernández-Cao et al. [13] conducted a systematic review and meta-analysis in order to evaluate the effect of dietary, supplementary, and total zinc intake and status on the risk of developing type 2 diabetes. This was based on the understanding that zinc may have a protective role against type 2 diabetes [13]. The results showed that a moderately high dietary zinc intake based on the ‘Dietary Reference Intake’ may reduce the risk of type 2 diabetes by 13% and up to 41% in rural settings [13]. In contrast, elevated serum/plasma zinc concentration was found to be associated with an increased risk of type 2 diabetes in the general population, although no relationship was established between total or supplementary zinc intake and type 2 diabetes [13]. Brandão-Lima et al. [14] also explored the relationship between the dietary intake of zinc, potassium, calcium, and magnesium and glycaemic control in patients with diabetes. The authors used multiple linear regression and binary logistic regression analysis to evaluate the effects of individual and combination intake of these micronutrients on glycated hemoglobin (HbA1c) and found a high likelihood of inadequate intake of the micronutrients. In addition, it was noted that the group with a lower micronutrient intake demonstrated higher % HbA1c (*p* = 0.006) and triglyceride (*p* = 0.010) levels [14].

Apart from evaluating the association between micronutrients and the risk of type 2 diabetes, the role of macronutrients and other metabolites in the development of this condition have been studied extensively. Song et al. [15] sought to examine whether dietary patterns that explain the variation of the triglyceride (TG) to high-density lipoprotein cholesterol (HDL-C) ratio were associated with the incidence of type 2 diabetes in Korean men and women. The authors found evidence that suggests that dietary patterns associated with low levels of TG/HDL-C ratio may have the potential to reduce the risk of type 2 diabetes.

Based on the above, it is essential that dietary management approaches that are tailored to meet the needs of people with type 2 diabetes reflect these elements that are aimed at reducing the risk of acute and chronic complications. In this regard, Hallberg et al. [9] noted in their narrative review that there is evidence that suggests the possible reversal of this condition through interventions and these have been incorporated into guidelines. These approaches may involve the use of bariatric surgery, low-calorie diets, or carbohydrate restriction [9]. In particular, the American Diabetes Association and the European Association for the Study of Diabetes have recommended a low carbohydrate diet and support the use of short-term low-calorie diets for weight loss.

A low carbohydrate diet (LCD), replacing some staple foods with nuts such as tree nuts and groundnuts, has been shown to reduce weight, improve blood glucose, and regulate blood lipid in patients with type 2 diabetes [16]. However, the consumption of tree nuts is difficult to promote in patients with diabetes because they are relatively more expensive compared to groundnuts [16]. It remains unclear whether peanuts and tree nuts, including almonds, in combination with LCD have similar benefits in patients with type 2 diabetes. Therefore, Hou et al. [16] conducted a randomized controlled trial to compare the effect of peanuts and almonds on the cardio-metabolic and inflammatory parameters in patients with type 2 diabetes. This was a parallel design involving 32 patients with type 2 diabetes [16]. The patients consumed a LCD with part of the starchy staple food being replaced with peanuts (peanut group) or almonds (almond group) and involved a follow-up period of three months [16]. The findings showed that the fasting blood glucose (FBG) and postprandial 2-h blood glucose (PPG) decreased in both the peanut and almond groups (*p* < 0.05) compared with the baseline, and, following the intervention, there was no significant difference between the peanut group and the almond group with respect to the FBG and PPG levels [16]. However, compared to the baseline value, there was a decrease in the glycated hemoglobin level in the almond group (*p* < 0.05) and no significant difference was found between the peanut and almond groups with respect to the HbA1c level at the third month. The authors concluded that when incorporated into a LCD, almonds and peanuts have a similar effect on improving fasting and postprandial blood glucose among patients with type 2 diabetes. 

In a separate study, Yamada et al. [17] conducted a systematic review of dietary approaches for Japanese patients with diabetes. The main focus of the review was to elucidate the effect of an energy-restricted and carbohydrate-restricted diet on the management of Japanese patients with diabetes [17]. All the randomized controlled trials included in the review showed better glucose management with the carbohydrate-restricted diet. It was found that carbohydrate-restricted diet, not the energy-restricted diet, might have short term benefits for managing Japanese patients with diabetes although the low number of studies included in the review was a limitation [17].

Burch et al. [18] also developed a protocol for a longitudinal study on evaluating how diet changes with the diagnosis of diabetes. It has been observed that the quality of diets plays a significant role in assisting people with type 2 diabetes to manage their condition and thus reduce the risk of developing diabetes-related complications [18,19]. This is because diet quality is the extent to which food intake complies with national or international dietary guidelines or a priori diet quality score and it influences glycaemic control in people with type 2 diabetes and has a significant impact on the risk of complications [18,20]. It often includes the macronutrient components of the diet. Thus, Telle-Hansen et al. [21] summarized the research evidence on randomized controlled trials of the effect of dietary polyunsaturated fatty acids (PUFAs) on glycaemic control in people with type 2 diabetes. This study was based on the fact that replacing saturated fatty acids (SFAs) with PUFAs decreases blood cholesterol levels and prevents cardiovascular diseases and that fat quality may also affect insulin sensitivity and increase the risk of type 2 diabetes [21]. Evidence from prospective studies has also shown that a high intake of SFAs can increase the risk of type 2 diabetes, while a high intake of PUFAs reduces the risk of the condition [21]. Based on this review, while about half of the studies that examined the effect of fish, fish oils, vegetable oils, or nuts found changes related to glycaemic control in people with type 2 diabetes, the other half found no effects [21]. In addition, it remains unclear whether PUFAs from marine or vegetable sources affect glycaemic regulation differently and this is a potential area for future research [21]. 

What is clear, however, is that a low glycaemic index (GI) diet is more effective in controlling glycated hemoglobin and fasting blood glucose than a high GI diet in patients with type 2 diabetes [22]. In a further systematic review and meta-analysis, Ojo et al. [23] sought to evaluate the effects of a low GI diet on the cardio-metabolic and inflammatory parameters in patients with type 2 diabetes and in women with gestational diabetes mellitus (GDM) and examine whether the effects are different in these conditions. While 10 randomized controlled studies were included in the systematic review, only 9 were selected for the meta-analysis [23]. The results of the meta-analysis found no significant differences (*p* > 0.05) between the low GI and higher GI diets with respect to total cholesterol, high-density lipoprotein (HDL), and low-density lipoprotein (LDL) cholesterol in patients with type 2 diabetes. With respect to the triglyceride, it increased by a mean of 0.06 mmol/L (0.01, 0.11) in patients with type 2 diabetes on a high GI diet and the difference compared with the low GI diet group was significant (*p* = 0.027) [23]. The results from the systematic review were not consistent in terms of the effect of a low GI diet on the lipid profile in women with GDM [23]. Furthermore, the low GI diet significantly decreased interleukin–6 (*p* = 0.001) in patients with type 2 diabetes compared to the high GI diet [23].

Nutritional approaches employed in managing patients with type 2 diabetes may also involve the use of enteral nutrition, including oral nutritional supplements (ONS) [3]. The effectiveness of these diabetes-specific formula (DSF) and standard formulas on glycaemic control and lipid profile in patients with type 2 diabetes continues to generate interest. Based on this, Ojo et al. [1] used a systematic review and meta-analysis of randomized controlled trials to evaluate the effect of diabetes-specific enteral nutrition formula on cardiometabolic parameters in patients with type 2 diabetes. On the other hand, Angarita Dávila et al. [3], conducted a randomized cross-over study to explore the effect of oral diabetes-specific nutritional supplements with sucromalt and isomaltulose compared with the standard formula (SF) on glycaemic index, entero-insular axis peptides, and subjective appetite in patients with type 2 diabetes. 

In the review by Ojo et al. [1], it was found that all the fourteen studies included in the systematic review showed that DSF was effective in lowering blood glucose parameters in patients with type 2 diabetes compared with SF. The results of the meta-analysis confirmed the findings of the systematic review with respect to the fasting blood glucose, which was significantly lower (*p *= 0.01) in the DSF group compared to SF, and the glycated hemoglobin, which was significantly lower (*p* = 0.005) in the DSF group compared to the SF group [1]. Based on the systematic review, the outcomes of the studies selected to evaluate the effect of DSF on lipid profile were variable. The authors concluded that the results provided evidence to suggest that DSF is effective in controlling fasting blood glucose and glycated hemoglobin and in increasing HDL cholesterol, but has no significant effect on other lipid parameters. They further noted that the presence of low glycaemic index (GI) carbohydrates, a lower amount of carbohydrates and a higher amount protein, the presence of mono-unsaturated fatty acids, and different amounts and types of fiber in the DSF compared with SF may be responsible for the observed differences in cardiometabolic parameters in both groups [1].

Angarita Dávila et al. [3] also compared the postprandial effects of oral diabetes-specific nutritional supplements with isomaltulose and sucromalt versus the standard formula (SF) on the glycaemic index (GI), insulin, glucose-dependent insulinotropic polypeptide (GIP), glucagon-like peptide 1 (GLP-1), and subjective appetite in people with type 2 diabetes. The subjects were given a portion of supplements containing 25 g of carbohydrates or reference food following overnight fasting [3]. The glycaemic index values were low for oral diabetes-specific nutritional supplements and intermediate for SF (*p* < 0.001). The area under the curve for insulin and GIP were lower (*p* < 0.02 and *p* < 0.02 respectively) after oral diabetes-specific nutritional supplements and higher (*p* < 0.05) for GLP-1 when compared with SF [3]. In addition, the subjective appetite area under the curve was greater (*p* < 0.05) after SF than oral diabetes-specific nutritional supplements [3]. 

The management of type 2 diabetes may also include the administration of insulin. But questions remain whether the dose of insulin before a meal should be based on glycemia or meal content [24]. Krzymien et al. [24] reviewed existing guidelines and scientific evidence on insulin dosage in people with type 1 and type 2 diabetes and explored the effect of the meal composition such as carbohydrate, protein and fat on postprandial glucose. The authors found that in most current guidelines aimed at establishing prandial insulin doses in type 1 diabetes, only carbohydrates are counted, whereas in type 2 diabetes the meal content is often not taken into consideration. Therefore, it was concluded that prandial insulin doses in managing people with diabetes should take into account the pre-meal glycemia, as well as the size and composition of the meals [24]. 

Apart from human studies, research based on the effects of different extracts on animal models have been conducted in an attempt to further elucidate our understanding of their role in diabetes. Tse et al. [25] assessed the glycemic lowering effect of an aqueous extract of *Hedychium coronarium* leaves in diabetic rodents. The study involved streptozotocin-induced type 2 diabetes Wistar rats and C57BKSdb/db mice. After treatment with *Hedychium coronarium* for 28 days, glucose tolerance improved in both of the diabetic animal models. The *Hedychium coronarium* also significantly improved the lipid profile in streptozotocin-induced type 2 diabetic rats [25]. On the other hand, Vlavcheski and Tsiani [26] explored the reduction of free fatty acid-induced muscle insulin resistance by Rosemary extract. It was found that Rosemary extract has the potential to counteract the palmitate-induced muscle cell insulin resistance [26].

In another study, Huang et al. [27] examined the effects of Tempeh fermentation with *Lactobacillus plantarum* and *Rhizopus oligosporus* on streptozotocin-induced type 2 diabetes rats. The results demonstrated that the modulation of serum glucose and lipid levels by lactic acid bacteria occurs via alterations in the internal microbiota, leading to the inhibition of cholesterol synthesis and promotion of lipolysis [27]. Furthermore, it was suggested that Tempeh, might be a beneficial dietary supplement for individuals with abnormal carbohydrate metabolism [27].

Yang et al. [28] also evaluated the combination of freeze-dried *Aronia*, red ginseng, ultraviolet-irradiated shiitake mushroom, and nattokinase in order to examine its effects on insulin resistance, insulin secretion, and the gut microbiome in a non-obese type 2 diabetic animal model. It was concluded that the combination of freeze-dried *Aronia*, red ginseng, ultraviolet-irradiated shiitake mushroom, and nattokinase improved glucose metabolism by potentiating insulin secretion and reducing insulin resistance in insulin-deficient type 2 diabetic rats [28]. The improvement of diabetic status ameliorated body composition changes and prevented changes in gut microbiome composition [28].

Overall, this editorial has demonstrated that a range of dietary interventions can provide useful approaches for managing people with type 2 diabetes including regulating blood glucose parameters and lipid profiles and for reducing the risks of acute and chronic diabetic complications.
